# Dynamic versus Static In Vitro Drug Release Testing of Subcutaneous Implants with Distinct Microstructures

**DOI:** 10.1021/acsomega.6c02143

**Published:** 2026-07-08

**Authors:** Scarlett Zeiringer, Laura Wiltschko, Bianca Brandl, Anbu Palanisamy, Thanh Nguyen, Matthias Katschnig, Martin Spoerk, Simone Eder, Eva Roblegg

**Affiliations:** † 27267 University of Graz, Institute of Pharmaceutical Sciences, Department of Pharmaceutical Technology and Biopharmacy, Universitätsplatz 1, 8010 Graz, Austria; ‡ 130347 Research Center Pharmaceutical Engineering GmbH, Inffeldgasse 13, 8010 Graz, Austria; § InnoCore Pharmaceuticals, L.J. Zielstraweg 1, 9713 GX Groningen, The Netherlands; ∥ Hage3D GmbH, Kratkystraße 2, 8020 Graz, Austria; ⊥ Institute of Process and Particle Engineering, Graz University of Technology, Inffeldgasse 13, 8010 Graz, Austria

## Abstract

Implantable long-acting drug delivery systems, such as subcutaneous implants, provide controlled, sustained release of an active ingredient. Although drug release kinetics are critical in the development of such systems, current in vitro release testing methods often fail to account for key physiological parameters such as pH, buffer capacity, flow conditions, as well as tissue/extracellular matrix (ECM) firmness and proteins. This study investigates the long-term in vitro release behavior of two biodegradable dexamethasone-loaded implants with different internal structures: dense implants produced via hot-melt extrusion (HME, 100% infill) and porous implants fabricated via fused filament fabrication (FFF, 25% infill). Phosphate buffers (10 mM and 100 mM), simulated body fluid, and simulated subcutaneous interstitial fluid were evaluated as potential release media. Among the tested buffers, the 100 mM phosphate buffer showed the highest long-term stability, maintaining a consistent buffer capacity with minimal fluctuations in pH and osmolarity and no precipitation at elevated temperatures. Protein-containing media, in general, showed precipitation and degradation and were therefore excluded from further studies. The effect of interstitial fluid flow was evaluated using the USP 4 apparatus, and release was studied over a period of 8 weeks. It was found that—compared to static conditions—drug release from porous FFF implants significantly increased from 23.71 ± 1.07% to 25.99 ± 0.41%. In contrast, dense HME implants exhibited diffusion-dominated release that was unaffected by flow. The underlying release mechanism, as determined by Korsmeyer–Peppas modeling, was dominated by diffusion for both implants, consistent with the behavior observed under static release conditions. The influence of tissue/ECM firmness was investigated in a gel-based setup using a 0.5% agarose gel, which allows diffusion of small molecules, mimics the bulk viscoelastic behavior of soft connective tissues/ECM, and shows comparable pore sizes. As expected, the overall drug release and underlying mechanism changed for both implant types, resulting in a lower absolute drug release and elevated *n*-values. Our findings provide fundamental insights into the interplay between relevant parameters, in particular flow and tissue/ECM firmness, and internal implant structures on the in vitro drug release, offering a framework to improve long-term testing of subcutaneous implants.

## Graphical abstract

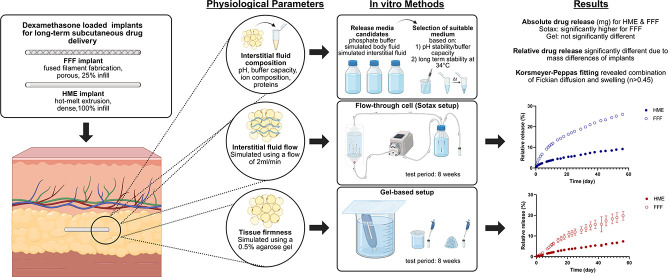

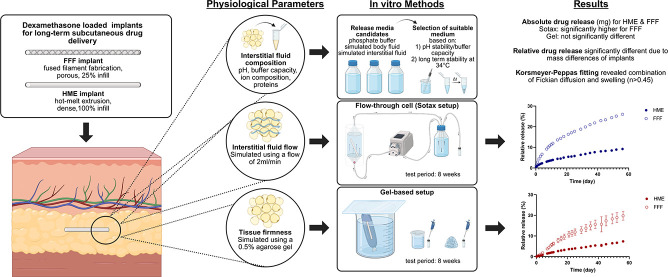

## 1 Introduction

Subcutaneous (SC) implants are a type of long-acting drug delivery system that has gained significant attention for their ability to achieve sustained drug release for weeks, months, or years. This approach is particularly beneficial for managing chronic diseases that require prolonged drug administration, as it improves patient compliance, maintains therapeutic drug levels over extended periods, and simplifies treatment regimens.^1,2^ However, accurately predicting drug release from such implants remains challenging due to the complex anatomical structure and highly dynamic nature of the SC environment.^3,4^

The subcutis consists of adipose and loose connective tissue, which produce extracellular matrix (ECM) components that provide mechanical strength.^5,6^ The ECM network, which fills the interstitial space, is composed of a collagen network and a gel-like matrix of glycosaminoglycans.^7^ The remaining interstitial space is perfused with interstitial fluid (ISF), along with blood and lymphatic vessels. Although ISF represents the primary biological environment encountered by SC drug formulations, its precise composition remains poorly characterized,^3,4^ as current isolation methods, such as suction blister methods or microdialysis, often alter its native state.^8^ Available data suggest that its protein composition resembles that of plasma but at lower concentrations, typically around 50%.^9,10^ The pH is comparable to that of plasma, ranging from 7.35 to 7.45.^4^ Thereby, the pH is regulated by the dissociation constant of water, the strong ion difference, and the partial tissue pressure of carbon dioxide (CO_2_). CO_2_ and weak acids, such as lactate, are byproducts of cellular metabolism, with CO_2_ buffered by blood equilibrium systems so it does not alter plasma pH, while weak acids are compensated by strong ions to maintain physiological pH.^11–13^ The key ions include sodium, present at the highest concentrations, and chloride, along with potassium, magnesium, calcium, (bi)­carbonate, sulfate, and negligible levels of phosphate.^14^ Besides ISF composition and pH, hydrostatic pressure is an important factor that influences drug dispersion and tissue fluid dynamics. This pressure depends on the elasticity of the ECM as well as the volume of fluid/solid introduced into the SC space, such as that delivered by injection or infusion. A more compliant ECM can accommodate higher fluid volumes with less pressure buildup, whereas a stiffer matrix may resist expansion and lead to elevated local pressures.^11,15^ Since the ECM in the SC region is generally soft and highly compliant,^6^ less pressure is exerted on solid implants. The temperature of the SC tissue is another important characteristic to be considered, as it is typically lower than the body core and ranges between 30 and 36 °C.^11,16^

Due to the growing importance of SC administration, a variety of formulations have been reported, including solutions, semisolids, particulate systems, and solid formulations. These allow for various release mechanisms and profiles, ranging from immediate and sustained delivery to stimuli-responsive release.^2,17^ Despite the broad range of SC formulations, there is a lack of in vitro release methods that consider the physiological conditions of the SC environment.^4^

Existing in vitro release methods used in the early-stage development of SC formulations are often adapted from systems originally designed for other dosage forms or rely on relatively simple, customized setups. USP device methods, such as the paddle method (USP apparatus 2) or flow-through cell (USP apparatus 4), are usually recommended by the FDA.^3,18,19^ With regard to the former, USP apparatus 2, implants or injectable microparticles are exposed to media under agitated conditions, considering sink conditions. The respective medium is either completely replaced or the removed volumes are replenished with fresh media. Alternatively, adjusted methods, such as dialysis bags, are used in the static or shaking mode.^3,18^ The USP apparatus 4 comprises flow-through cells, a media reservoir, and a pump, and can be run in open- or closed-loop configurations.^20,21^ In an open-loop setup, the medium flows through the cell once before being directed to a detector or discarded. This setup typically demands a large volume of medium. In contrast, a closed-loop system recirculates the medium through the cell throughout the entire duration of the test, with samples taken from the reservoir at regular intervals and replaced with fresh medium.^22^ Although the flow rates are low under SC conditions, release rates, particularly of porous systems under long-term studies, are likely to be affected by the nonstatic release conditions, flow, and convective transport.^23,24^ The release media in the experiments commonly include simple buffers like phosphate-buffered saline (PBS),^3^ Hank’s balanced salt solution,^25,26^ simulated body fluid (SBF),^27,28^ and SC interstitial buffer (SIB).^29^ While some of these models have demonstrated correlations between in vitro release and in vivo absorption in specific cases,^30^ such outcomes are not necessarily transferable to other drug formulations. More advanced systems that include simplified ECM matrices in addition to the corresponding buffer systems are the Emulator of SubCutaneous Absorption and Release (ESCAR) system,^31,32^ the Subcutaneous Injection Site Simulator (SCISSOR),^33–35^ and a newly developed add-on for the USP 4 Sotax system, the BioJect cell.^36^ These systems enable the investigation of drug diffusion and absorption.

To evaluate the influence of static and dynamic dissolution methods on the release of long-acting dexamethasone (DEX)-loaded SC implants with different infills (nonporous hot-melt extrusion (HME) implants and porous fused filament fabrication (FFF) implants), several factors were considered within this study. In the first step, the long-term stability of different buffer systems (taking the implantable devices into account) was investigated with regard to buffer capacity, ionic strength, pH, and osmolarity, both with and without protein. In the second step, the most stable buffer system was selected, and the influence of ISF flow on DEX release from both implants was studied. To this end, a Sotax flow-through system was used, and data were generated over a period of 8 weeks. To simulate the firmness of the SC tissue/ECM, additional release studies were conducted using a modified gel system (0.5% agarose). Finally, the results were compared to release data obtained from a static setup by Brandl et al.,^37^ and the key factors influencing the release mechanism of DEX were evaluated.

## 2 Materials and Methods

### 2.1 Materials

The following reagents were used in this study: micronized DEX (d90 < 10 μm) was obtained from Shenzhen Nexconn Pharmatechs Ltd. (Shenzhen, China). Human serum albumin solution (Albunorm 200 g/L) was purchased from Octapharma Pharmazeutika (Vienna, Austria). Agarose (low melting point, gel strength ≥ 200 g/cm^2^, congealing temperature 26–30 °C, CAS: 39346–81–1) was obtained from Sigma-Aldrich (Munich, Germany). Acetonitrile (ACN) and formic acid were sourced from Honeywell International Inc. (Morristown, NJ, USA). Trifluoroacetic acid (≥99.0%), hydrochloric acid (37%), sodium chloride (NaCl), potassium chloride (KCl), sodium acetate (CH_3_COONa), and sodium sulfate decahydrate (Na_2_SO_4_·10H_2_O) were purchased from Carl Roth GmbH & Co. KG (Karlsruhe, Germany). Potassium dihydrogen phosphate (KH_2_PO_4_) and disodium hydrogen phosphate (Na_2_HPO_4_) (Ph. Eur., ACS) were obtained from VWR Chemicals (Radnor, PA, USA). Dipotassium hydrogen phosphate (K_2_HPO_4_), magnesium chloride hexahydrate (MgCl_2_·6H_2_O), sodium bicarbonate (NaHCO_3_), and calcium chloride dihydrate (CaCl_2_·2H_2_O) were purchased from Merck KGaA (Darmstadt, Germany). Water was purified using a Milli-Q Water (MQ) Purification System (Merck KGaA, Darmstadt, Germany). Biodegradable poly­(ether ester) multiblock copolymers (brand name 20LP10L20-GLL40) from the SynBiosys platform (InnoCore Pharmaceuticals, Groningen, Netherlands), based on poly­(d,l-lactic acid)–poly­(ethylene glycol)–poly(d,l-lactic acid) (PDLLA–PEG–PDLLA) and poly­(glycolide-*co*-l-lactide) (PGLLA) blocks, were used for implant fabrication. Further information on the used polymers is provided elsewhere.^38^

### 2.2 Implant Design

The design of the implants was based on the geometry of the commercial SC implant Nexplanon, which is a cylindrical rod with a diameter of 2 mm and a length of 40 mm.^39^ To evaluate the impact of implant design on drug release kinetics, two variants were fabricated: a solid, nonporous implant produced via HME, and a porous implant manufactured by FFF using a 25% rectilinear infill density and no perimeter.

### 2.3 Implant Fabrication

Drug-free and drug-loaded biodegradable polymer extrudates (2 mm diameter, 35% DEX (w/w)) and drug-loaded filaments (1.75 mm diameter, 35% DEX (w/w)) were prepared via HME according to a published protocol by Brandl et al.^37^ For the preparation of the extruded implants (HME implants), the 2 mm diameter extrudates were cut into 40 mm rods, yielding solid, nonporous monolithic implants. 3D printing was conducted using an FFF printer (Medmex, Hage3D GmbH, Graz, Austria) and Simplify3D V5.0.2 (Simplify3D, Cincinnati, OH, USA) as the slicer software. Cylindrical implants (2 mm diameter × 40 mm length, geometry slightly adapted via the software AutoCAD (Autodesk, San Rafael, CA, USA) to guarantee first-layer adhesion during FFF,^37^ 25% infill density with no outer perimeter) were printed from the filament, creating an open porous internal structure (FFF implant) (Figure 1). Printing was performed using a 250 μm nozzle, a printing speed of 10 mm/s, and a nozzle temperature of 170 °C.

**Figure 1:**
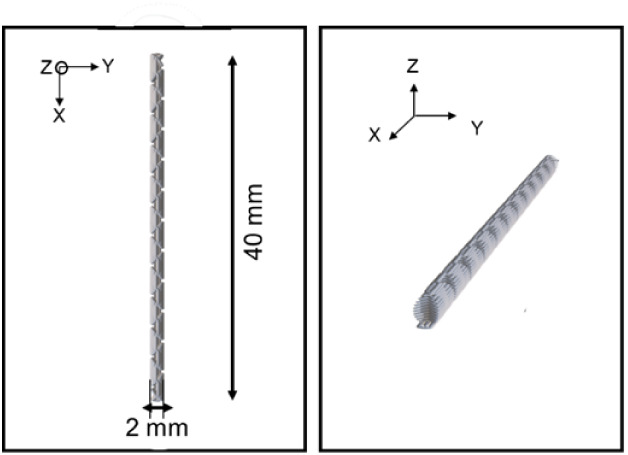
3D design of the 3D-printed porous implant.

### 2.4 Buffer Preparation

Four different buffer systems were prepared: phosphate buffer (PB) 10 mM and 100 mM, SBF, and simulated subcutaneous interstitial fluid (SSIF). The PB was prepared by dissolving Na_2_HPO_4_ and KH_2_PO_4_ in Milli-Q water under constant stirring. Subsequently, the buffer was heated to 34 °C, and the pH was adjusted to 7.4 ± 0.1. SBF and SSIF were produced according to the protocols described by Kokubo et al.^40,41^ and Gao et al.^29^ (Table 1) with minor adjustments. The preparation of the buffers involved the sequential dissolution of NaCl, NaHCO_3_, KCl, K_2_HPO_4_, MgCl_2_, CaCl_2_, Na_2_SO_4_, and CH_3_COONa (for SSIF only) in Milli-Q water (37 °C) under continuous stirring. Following the complete dissolution of all components, the solution was equilibrated at 34 °C, and the pH was adjusted to 7.4 ± 0.1. For SSIF, HSA was added to a final concentration of 2%, followed by pH measurement and further adjustment to pH 7.4 ± 0.1, if necessary. A lower concentration was chosen compared to the reported 25 g/L FBS in SSIF^29^ as the focus was solely on the main binding protein HSA, and 2% HSA has already been used to simulate ISF.^42,43^ All buffer systems were stored at 4 °C and used in experiments within a maximum of 2 days.

**Table 1:** Ion (mM) and Protein (g/L) Concentrations of the Prepared Phosphate Buffers (PB), Simulated Body Fluid (SBF), and Simulated Subcutaneous Interstitial Fluid (SSIF) Compared to Human Interstitial Fluid (ISF) Data Obtained from the Literature

	10 mM PB (mM)	100 mM PB (mM)	SBF (mM)	SSIF (mM)	Human ISF (mM)^29^
Na^+^	16.3	163.4	142	136	143
K^+^	1.8	18.2	5	3.9	4
Mg^2+^	–	–	1.5	0.5	0.7
Ca^2+^	–	–	2.5	1.3	1.3
Cl^–^	–	–	147.8	114.9	115
SO_4_^2–^	–	–	0.5	0.5	0.5
HCO_3_^–^	–	–	4.2	20.6	28
H_2_PO_4_^–^	1.8	18.2	–	–	–
HPO_4_^2–^	8.2	81.7	1	1	1
CH_3_COO^–^	–	–	–	5	5.0
Protein	–	–	–	20 g/L HSA	26 g/L

### 2.5 Buffer Characterization: pH, Osmolarity, Stability, and Drug–Protein Binding

For buffer characterization, the pH and osmolarity of all buffer systems were measured after incubation with the biodegradable polymer. For this, drug-loaded HME implants were incubated in glass bottles containing 3 and 10 mL of PB, SBF, and SSIF for up to 7 days at 34 °C and 50 rpm in an incubator shaker. At predetermined time points (i.e., after 1 h, 2 h, 4 h, 6 h, 1 d, 2 d, 3 d, and 7 d), the pH (Mettler Toledo GmbH, Greifensee, Switzerland) and osmolarity (OSMOMART 030-D automatic cryoscopic osmometer; Gonotec, Berlin, Germany) were determined (*n* = 3). For the 3 mL samples, the buffer was fully exchanged at each measurement point; the 10 mL buffers were not replaced during the entirety of the test period.

Furthermore, the stability of SBF and SSIF was examined over a period of 10 days. The buffers were incubated at 4 °C, 25 °C, and 34 °C, and the pH value and osmolarity were measured once a day. In addition, the buffers were visually inspected for precipitation. The experiment comprised four distinct settings: 1) buffer only, 2) buffer spiked with 8 μg/mL DEX (equivalent to approximately 10% of the saturation concentration), 3) buffer with a drug-free HME implant, and 4) buffer with a drug-loaded HME implant.

Drug–protein binding of DEX to its primary binding partner, HSA, was evaluated via rapid equilibrium dialysis (RED) using a protocol adapted from Wiltschko et al.^43^ (Supporting Information).

### 2.6 Gel Preparation and Characterization

The gel was chosen based on previous literature to mimic SC tissue.^44–48^ For gel preparation, agarose powder was added to preheated 100 mM PB (100 °C) and stirred for 10 min until fully dissolved, and then poured into the respective containers or dialysis bag (Spectra/Por 7, MWCO 1 kDa, with a diameter of 45 mm) after cooling to 40–45 °C.

The density of the 0.5% (w/v) agarose gel was measured using the DAS 5000 M density and sound velocity meter (Anton Paar GmbH, Graz, Austria) (*n* = 3). Rheological characterization of the freshly prepared gel was performed using the Physica MCR 301 rotational rheometer (Anton Paar GmbH, Graz, Austria) equipped with a PP08 parallel plate system. Briefly, 150 μL of dissolved agarose solution was pipetted into custom-designed 3D-printed cylindrical molds (12 mm in diameter) and allowed to gel at room temperature under a protective cover for 1 h. All experiments were performed at 34 °C, and the measurement gap was set to 1.75 mm. The linear viscoelastic (LVE) region of the gel was determined via an amplitude sweep over a deformation range (γ) of 0.1% to 100% at a constant angular frequency of 10 rad/s. Following the identification of the LVE, a strain of 0.1% was selected for frequency sweep measurements, which were conducted over an angular frequency range of 0.1 to 500 rad/s. Additionally, the dynamic viscosity was measured as a function of shear rate from 0.1 to 100 1/s.

### 2.7 Polymer Swelling

The swelling behavior of the drug-loaded HME implant was investigated gravimetrically according to the protocol by Brandl et al.^37^ over 3 weeks under fluid flow and embedded in a 0.5% agarose gel. To simulate flow conditions, the implants were placed in a Sotax system (Sotax CE7 Smart USP Apparatus 4, Aesch, Switzerland) as described in Section 2.8. Briefly, 100 mM PB was circulated in the system at 2 mL/min and 34 °C. In the gel setup, the implants were embedded in 40 mL of 0.5% agarose gel (prepared with 100 mM PB at pH 7.4; see Section 2.9) and incubated at 34 °C under static conditions. At predetermined time points (i.e., 2, 4, and 8 h, followed by 24 h intervals excluding weekends), the implants were carefully removed from the system. Any residual surface fluid/gel was gently dabbed off, and the implants were weighed using an analytical balance. The relative mass was calculated by normalizing to the initial dry mass (defined as 100%). Measurements were terminated when no further mass increase was observed. All measurements were performed in triplicate. The swelling behavior was only investigated for the HME implants, as for the FFF implants, the removal of excess fluid or gel in the porous network was challenging and led to highly fluctuating results.

### 2.8 Drug Release Studies—Sotax USP 4

The DEX release from HME and FFF implants was investigated using a Sotax CE7 Smart USP apparatus 4 dissolution system equipped with an IPC-8 syringe pump (ISMATEC, Sotax, Aesch, Switzerland) and a C615 autosampler. The system was operated in a closed-loop configuration, with 100 mM PB (pH 7.4) at 34 °C serving as the drug release medium. The samples (*n* = 3) were placed in 22.6 mm dissolution cells (14 mL), whereby the cone was filled with glass beads (1 mL) to ensure laminar flow and adjust the volume (Figure 2). The total dissolution media volume for each implant was 300 mL. The flow rate and sample volume were set to 2 mL/min and 1 mL, respectively. At predetermined points, samples were withdrawn by the autosampler and analyzed via HPLC-UV. To maintain sink conditions, i.e., a maximum of 10% of the DEX saturation solubility (i.e., 76 ± 2 μg/mL, measurements done according to USP 1236 by Brandl et al.^37^), parts of the dissolution medium were exchanged with preheated 100 mM PB (34 °C) at every sampling point. Overall, testing was performed over 8 weeks, and samples were stored at −20 °C until further analysis.

**Figure 2:**
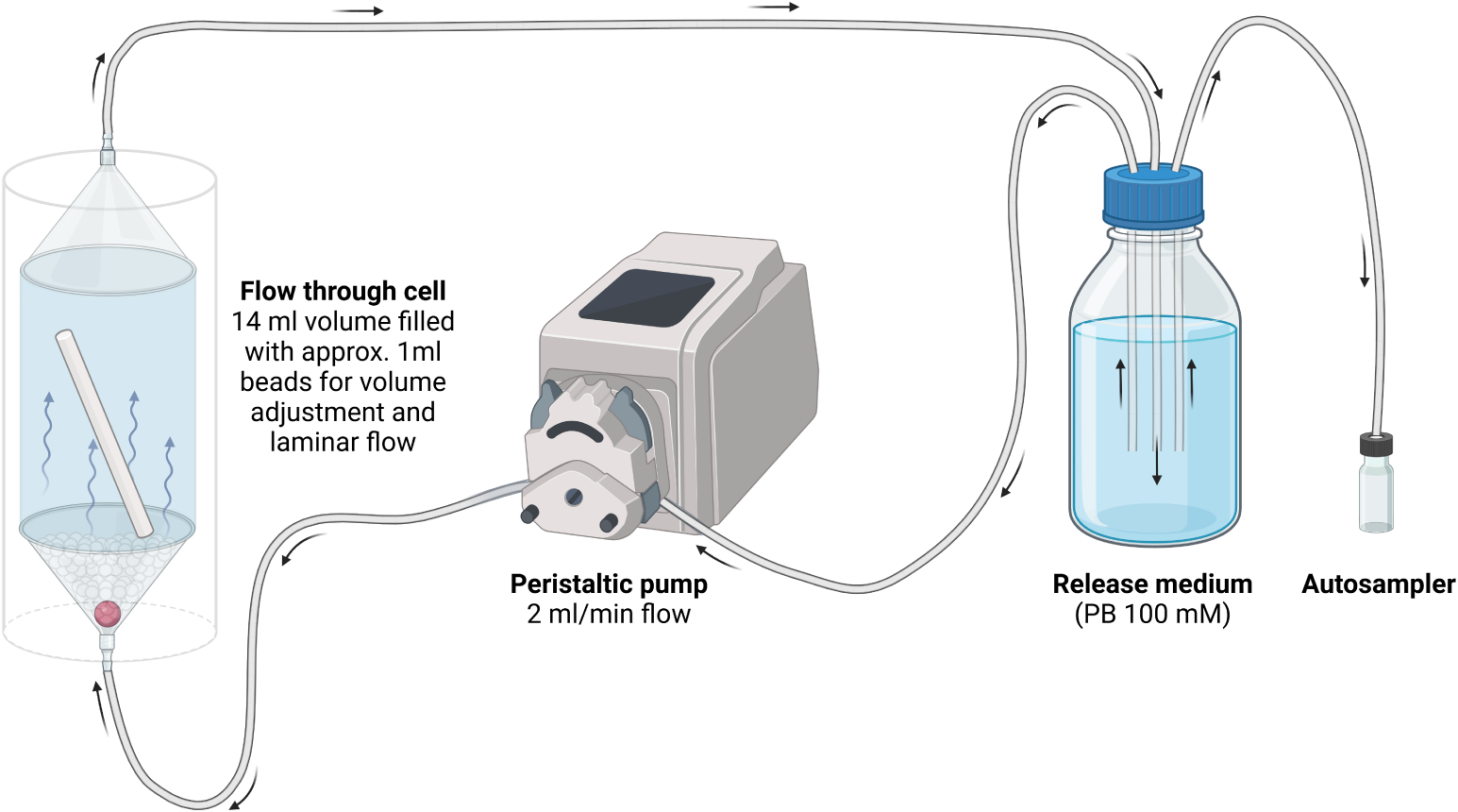
Schematic setup of the Sotax USP 4 system used for the in vitro release testing of the implants under dynamic conditions.

### 2.9 Drug Release Studies—Gel Setup

To simulate drug release from the implants under more physiologically relevant in vitro testing conditions, the drug release from gel-embedded implants was investigated. To this end, a 0.5% agarose gel prepared with 100 mM PB and 40 mL was filled into dialysis tubes (Spectra/Por 7, MWCO 1 kDa, with a diameter of 45 mm). A 3D-printed scaffold containing the fixed HME and FFF implants (*n* = 3) (Figure 3) was used for stabilization and reproducible placement of the implants. The tube was then closed and placed in 300 mL of 100 mM PB at 34 °C. To investigate the drug release, 1 mL samples was drawn from the buffer at predetermined time points equal to those in Sotax studies (Section 2.8). Specific buffer volumes were exchanged at each sampling point to maintain sink conditions. Additionally, the DEX concentration in the agarose gel was determined after 8 weeks. Prior to DEX quantification by HPLC-UV, the gel was extracted from the dialysis tube and homogenized. Then, 1 mL of the gel was mixed with 1 mL PB/EtOH (1:1, v/v) and filtered through a PVDF syringe filter (pore size 0.45 μm) to remove any solid components. Again, testing was performed over 8 weeks, and all samples were stored at −20 °C prior to further analysis.

**Figure 3:**
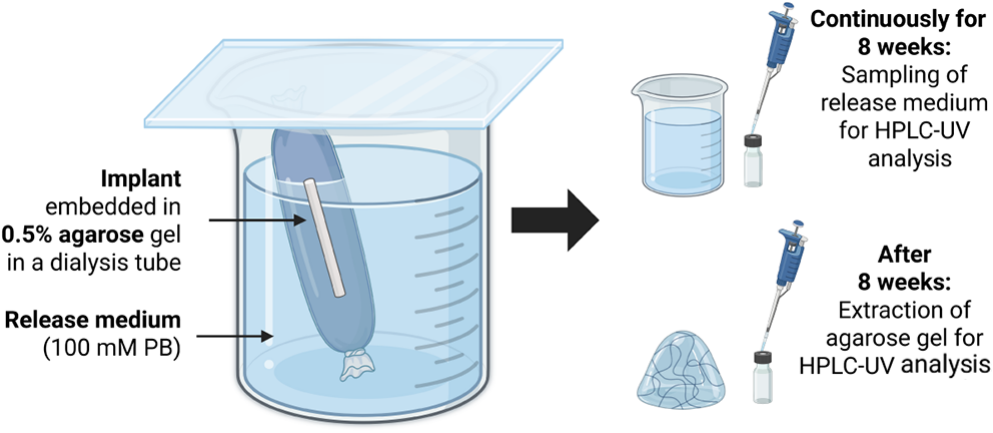
Schematic of the gel-based setup used for testing the drug release of the implants surrounded by a 0.5% agarose gel mimicking tissue firmness.

### 2.10 DEX Quantification via HPLC-UV

Quantification of DEX was performed by HPLC-UV using a Waters Acquity H-Class System (Waters GmbH, Eschborn, Germany). The system was equipped with an Acquity UPLC column (BEH C18, 1.7 μm, 2.1 mm × 50 mm; Waters GmbH, Eschborn, Germany) and operated at 30 °C. The separation was performed in isocratic mode with a flow rate of 0.35 mL/min and an injection volume of 2 μL. As the mobile phase, trifluoroacetic acid in water (0.05% v/v) and ACN in a ratio of 7:3 (v/v) were used. Sample analysis was performed at 240 nm. The linearity of the method was confirmed in the concentration range from 1 to 200 μg/mL in PB. For the gel samples, calibration concentrations ranged from 0.5 to 30 μg/mL DEX in 0.5% agarose gel. Sample preparation was performed as described in Section 2.9.

### 2.11 Release Data Fitting and Statistics

Results are shown as the mean and standard deviation. Significance was tested using a two-tailed Mann–Whitney U test for independent groups. Statistical significance was indicated as follows: * *p*<0.05, ** *p*<0.01, *** *p*<0.001, and **** *p*<0.0001.

The drug release mechanisms from the implants were evaluated via fitting the release data according to the Korsmeyer–Peppas model^49,50^ described by eq. 1,

$$\frac{M_{t}}{M_{\infty }}=kt^{n}$$

where *M_t_*/*M*_∞_ is the DEX mass released at time *t* divided by the mass released at infinite time (i.e., the drug loading mass). In other words, *M_t_*/*M*_∞_ is the fraction of DEX released. Here, *k* is the constant accounting for structural and geometrical characteristics, *t* is the release time, and *n* is the release exponent, which indicates the underlying mechanism of drug release. For cylindrical systems, an *n* of 0.45 indicates Fickian diffusion, and an *n* of 0.89 indicates zero-order release. Values in between are due to anomalous transport.

## 3 Results

### 3.1 Implant Fabrication via HME and FFF

Table 2 shows the key parameters (i.e., weight and drug loading) of the implants prepared by HME and FFF. Both methods were used to produce implants with the same external dimensions but different internal structures. Consequently, the implants differed in total weight (i.e., HME ∼ 150 mg vs FFF ∼ 60 mg) with a targeted drug loading of 35% DEX. Complete characterization of the implants is provided in the publication by Brandl et al., including the measured drug loading of 36.4 ± 1.2% and 36.4 ± 2.8% for the HME implant and the FFF implant, respectively.^37^

**Table 2:** Key Parameters of the Hot-Melt Extrusion (HME) and Fused Filament Fabrication (FFF) Implants, Including Implant Weight (mg) and Absolute Drug Loading (mg)

	HME implant	FFF implant
Weight (mg)	152.2 ± 2.0	58.7 ± 1.8
Absolute drug loading (mg)	55.4 ± 0.7	21.4 ± 0.6

### 3.2 Buffer Characterization

For 100 mM PB, SBF, and SSIF, pH fluctuations in both setups, i.e., 3 and 10 mL, were approximately ±0.2, with minor differences between buffer systems. However, it is important to note that 10 mM PB had been excluded from further detailed analysis in prior studies due to its pronounced pH shifts under similar experimental conditions (data not shown). After polymer incubation, all buffer systems, except the 10 mL 100 mM PB setup, showed a small but significant decrease in pH over 7 days (Supporting Information). Similarly, control setups without incubated polymer exhibited a small but significant decrease in pH for SBF and SSIF. Overall, the observed changes were comparable to those in the controls, indicating that polymer incubation had no significant impact on the pH. Given the low absolute changes in pH, these effects are negligible in practice. When comparing the mean pH over 7 days, 100 mM PB showed the smallest variation of all setups (Supporting Information).

Osmolarity significantly increased over time in all setups (Supporting Information). In general, 100 mM PB showed a lower osmolarity compared to SBF and SSIF. Notably, in the 3 mL setup, the strongest increase in osmolarity after 7 days was observed with SSIF (+34.15 ± 0.13%), followed by SBF (+24.53 ± 7.62%), while the smallest increase occurred in 100 mM PB (+13.96 ± 7.64%). In the 10 mL setups, the increase was comparable to the control setups (Supporting Information).

Stability of SBF and SSIF (pH and precipitation) was assessed in four setups (buffer alone; buffer with DEX; buffer with drug-free HME implant; buffer with drug-loaded HME implant) at 4 °C, 25 °C, and 34 °C. For all temperatures, the buffers showed pH fluctuations similar to those in the previously reported 10 mL setup, with the lowest pH fluctuations at 4 °C. Visual observations indicated precipitation in SBF and SSIF at 25 and 34 °C starting on day 6, while the buffers remained stable at 4 °C. This occurred independently of polymer or DEX presence and time. Due to the stability issues with SBF and SSIF, 100 mM PB was chosen for all further experiments.

RED experiments showed a ∼50% protein-bound DEX fraction in the presence of 2% HSA (Supporting Information). However, due to stability issues, no protein-containing media were used for the long-term release studies.

### 3.3 Gel Characterization

The 0.5% agarose gel showed a mean density of 1.008 ± 0.001 g/cm^3^. Rheological studies revealed a predominantly elastic behavior over the tested frequency and shear rate ranges. In the frequency sweep, the storage modulus (G′) was consistently higher than the loss modulus (G″) across all measured frequencies (Figure 4a), confirming the formation of a stable gel network with solid-like characteristics. G′ remained relatively constant in the low-frequency region (∼10^3^ Pa), while G″ was significantly lower (∼10 Pa), indicating that the gel’s elastic contribution dominates the viscous response. The complex viscosity (η*) measured over the same frequency range exhibited a decrease with increasing angular frequency (Figure 4b), reflecting a typical shear-thinning behavior. Steady shear measurements (0.1 to 25 s^–1^) further confirmed this, showing a progressive reduction in apparent viscosity at higher shear rates (Figure 4c). 100 mM PB was used for the preparation to ensure physiological pH and defined molarity.

**Figure 4:**
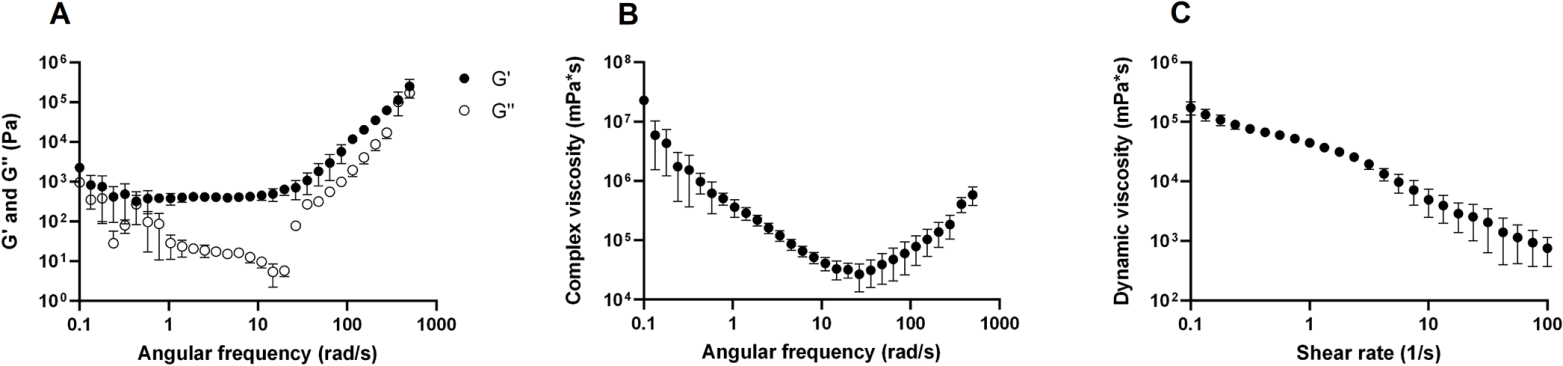
Rheological evaluation of the 0.5% agarose gel: A) Storage (G′) and loss modulus (G″) obtained from frequency sweep measurements. B) Complex viscosity obtained from frequency sweep measurements. C) Dynamic viscosity.

### 3.4 Implant Swelling

The swelling capacity of the polymer was evaluated in 100 mM PB under dynamic conditions using the Sotax USP4 system, as well as in the gel setup employed for the drug release studies. As shown in Figure 5, the HME implant exhibited significantly faster water uptake when embedded in the 0.5% agarose gel (*p =* 0.0476*) compared to the Sotax setup. These differences were already evident after 4 h of incubation (*p =* 0.0018**) and resulted in a swelling capacity of 108.9 ± 0.7% in the gel setup and 104.2 ± 0.3% in the Sotax setup after 21 days, starting from the initial implant weight (=100%).

**Figure 5:**
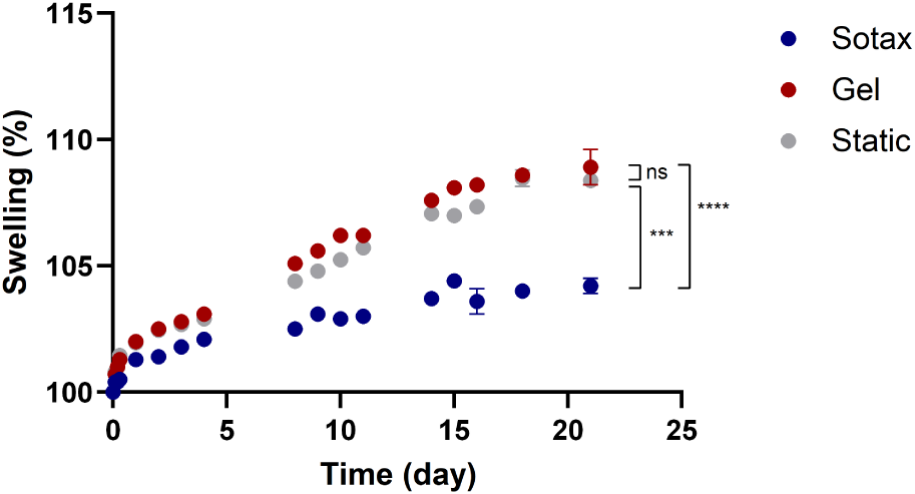
Swelling capacity (%) of the hot-melt extrusion (HME) filament in the dynamic Sotax setup and the gel setup compared to swelling data in a static release setup by Brandl et al.^37^ Significance was tested for the swelling capacity at day 21. Static vs Sotax = **** (*p* < 0.0001); Static vs Gel = ns (*p* = 0.2853); Sotax vs Gel = *** (*p* = 0.0004).

### 3.5 Drug Release Studies

On the basis of the results of the stability tests, 100 mM PB was used as the medium for the drug release studies. No HSA was added to the release medium due to issues with stability over the planned study duration of 8 weeks. The results were compared with DEX-loaded implants from a static release study conducted by Brandl et al.^37^

Under dynamic conditions, 5.06 ± 0.21 mg DEX was released from the HME implant compared to 5.56 ± 0.09 mg DEX from the FFF implant after 8 weeks (*p* = 0.0183*) (Supporting Information–Figure S1). This corresponds to a relative cumulative release of 9.19 ± 0.28% and 25.99 ± 0.41%, respectively. In contrast, lower values were observed in the gel setup, where drug concentrations above the LLOQ of the HPLC-UV method were only found after 1 day. The absolute cumulative DEX release after 8 weeks found in the surrounding PB was 4.11 ± 0.15 mg for HME and 4.19 ± 0.26 mg for FFF implants (*p* = 0.6513) (Supporting Information–Figure S1), translating to a relative release of 7.44 ± 0.31% and 19.97 ± 1.93%, respectively. Additionally, the DEX concentration present within the surrounding agarose gel was analyzed, as it contributes to the total amount of drug released. After 8 weeks, the gel exhibited DEX concentrations of 5.16 ± 0.68 μg/mL for HME and 5.53 ± 0.89 μg/mL for FFF implants, corresponding to an additional 0.21 ± 0.03 mg and 0.22 ± 0.04 mg of released DEX, respectively. Overall, a total release of 4.32 ± 0.15 mg and 4.41 ± 0.26 mg for the HME and FFF implants was measured in the gel setup, which is significantly lower than in the Sotax setup (HME: *p* = 0.0077**; FFF: *p* = 0.0019**). With regard to the daily DEX release, a considerably higher release was observed in the Sotax setup during the first 3 days in comparison to the gel setup. After day 4, no substantial differences were detected between the two conditions (Figure 6).

**Figure 6:**
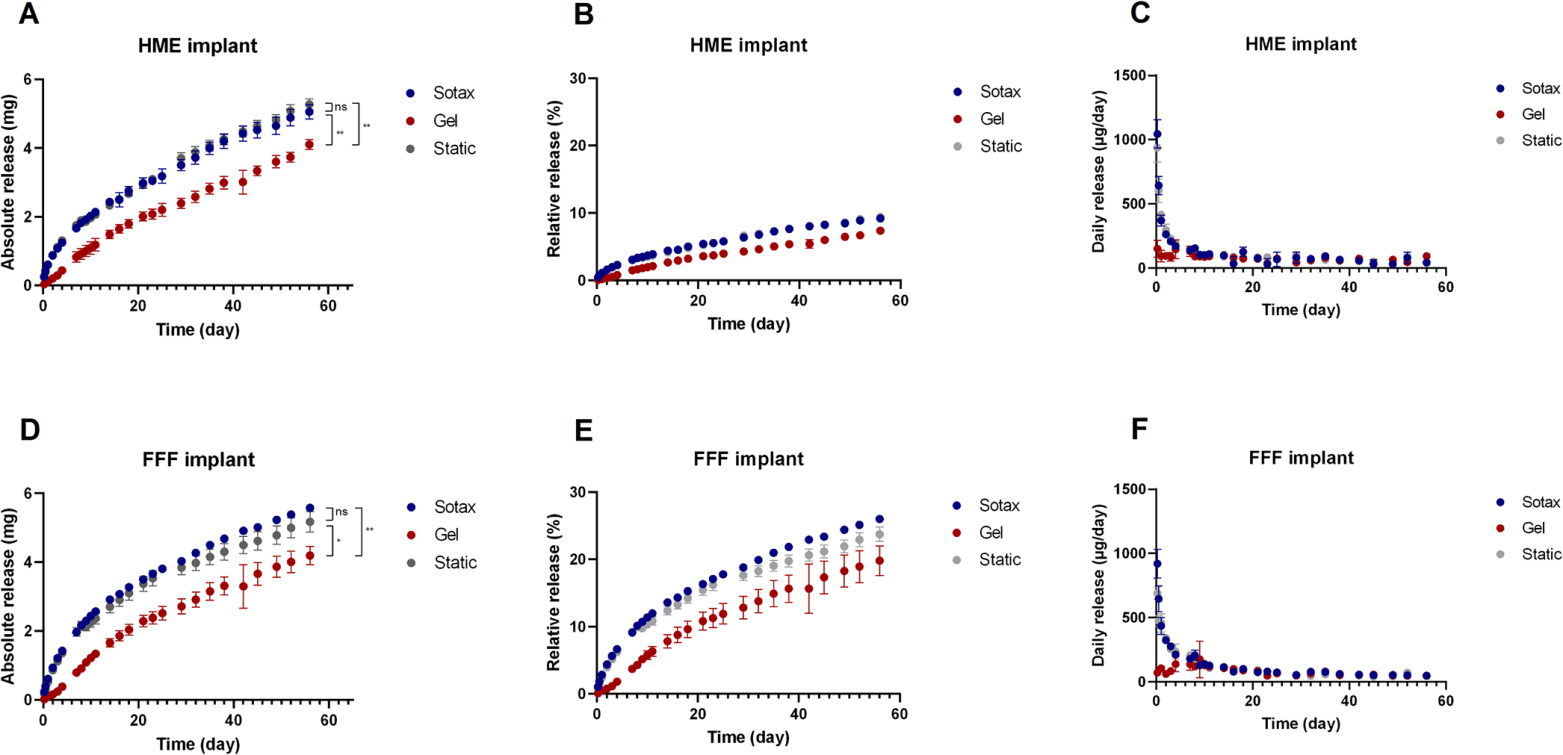
Drug release profiles of the hot-melt extrusion (HME) and fused filament fabrication (FFF) implants in the Sotax setup and the gel setup over 8 weeks compared to data obtained in a static release setup by Brandl et al.^37^ A) Absolute drug release (mg) of the HME implant. B) Relative release (%) of the HME implant. C) Daily drug release (μg/day) of the HME implant. D) Absolute drug release (mg) of the FFF implant. E) Relative release (%) of the FFF implant. F) Daily drug release (μg/day) of the FFF implant. Significance was tested for the cumulative absolute drug release at day 56. (A) HME: Static vs Sotax = ns (*p* = 0.2416); Static vs Gel = ** (*p* = 0.0018); Sotax vs Gel = ** (*p* = 0.0077). (D) FFF: Static vs Sotax = ns (*p* = 0.0948); Static vs Gel = * (*p* = 0.00282); Sotax vs Gel = ** (*p* = 0.0019).

The drug release profiles of the HME and FFF implants were fitted using the Korsmeyer–Peppas model to evaluate the release mechanism in different setups and time intervals. The results, including the release exponent *n*, release rate constant *k*, and coefficient of determination (*R* ^*2*^), are summarized in Table 3. For the Sotax setup, the initial phase (0.25–2 days) of both the HME and FFF implants exhibited *n*-values of 0.550 and 0.645, respectively. These values fall within the range typically associated with anomalous (non-Fickian) transport, which implies a combined influence of diffusion and polymer relaxation or swelling. In the subsequent release phase (3–56 days), *n-*values exhibited a slight decrease to 0.538 (HME) and 0.491 (FFF), suggesting a shift toward more diffusion-dominated release with residual anomalous characteristics. In the gel setup, a markedly different release behavior was observed, with a later onset of the initial phase as samples before day 1 were below the LLOQ. During the initial phase (1–7 days), both HME and FFF implants demonstrated *n*-values greater than 1 (1.140 and 1.285, respectively), indicating Super Case II transport. In the later phase (8–56 days), *n*-values declined to 0.747 (HME) and 0.698 (FFF), indicating the presence of anomalous transport mechanisms, where both diffusion and polymer matrix dynamics govern the release. In the Sotax and the gel setups, the FFF implant showed higher *k*-values compared to the HME implant. When comparing both, consistently higher *k*-values for all time frames were obtained in the Sotax setup. Additionally, all *R* ^2^ values were ≥0.995, which indicates a good fit of the model.

**Table 3:** Kinetic Parameters Obtained from Korsmeyer–Peppas Model Fitting, Including the Respective Time Frame of the Drug Release Phase, the Kinetic Constant (*k*), the Diffusional Release Exponent (*n*), and the Coefficient of Determination (*R* ^2^)

	Time frame (d)	*k* (d^–1^)	*n*	*R* ^2^	Release phase
HME implant	0.25–2	0.011	0.550	0.997	Anomalous (early phase)
Sotax	3–56	0.011	0.538	0.998	Fickian diffusion dominated
FFF implant	0.25–2	0.028	0.645	0.999	Anomalous (early phase)
Sotax	3–56	0.036	0.491	0.998	Fickian diffusion dominated
HME implant	1–7	0.002	1.140	0.997	Super case II
Gel	8–56	0.004	0.747	0.997	Anomalous (mixed)
FFF implant	1–7	0.003	1.285	0.995	Super case II
Gel	8–56	0.012	0.698	0.995	Anomalous (mixed)

## 4 Discussion

The use of biodegradable, long-acting implants is becoming increasingly important, as they enable sustained drug release over periods of weeks to months. To control drug release, implants with different designs are required, which can result in distinct release profiles. Long-term release studies, therefore, require, on the one hand, stable buffer systems that maintain physiological properties such as pH, buffer capacity, and osmolality, and, on the other hand, account for stress factors such as ISF flow and the stiffness of the tissue/ECM.^24^ These factors can play a particularly significant role when comparing 3D-printed porous implants with dense ones.

For all tested buffer systems, the observed pH fluctuations of approximately ±0.2 remained within a physiologically tolerable range. Such minor shifts are likely driven by interactions with atmospheric CO_2_ and O_2_, leading to acid–base disturbances through carbonate buffering and oxidation/reduction reactions,^51^ particularly in SBF, SSIF, and 10 mM PB. In vivo, such fluctuations would be counteracted by intrinsic ISF buffering components, such as bicarbonate, phosphate, and proteins, which stabilize pH and maintain tissue homeostasis.^52^ Interestingly, neither SBF nor SSIF exhibited greater pH stability compared to PB, despite their more physiological, ISF-like ion compositions. This can be attributed to the higher concentration of buffering species in 100 mM PB and suggests that buffer capacity is a more decisive factor in maintaining pH stability than replicating the complex ionic composition of ISF. It is also notable that increasing the salt concentration in PB from 10 mM (commonly used, e.g., refs. 53–56 ) to 100 mM significantly enhanced the pH stability, which is critical for a reliable assessment. This improvement is particularly relevant for systems that release acids or bases, such as polymers undergoing hydrolytic degradation, as they can substantially influence the pH of the release medium.^24^ In vivo, ISF pH is maintained within a narrow range due to its inherent buffering capacity and continuous exchange with the vascular system, where blood provides robust pH regulation.^57^ Therefore, the in vitro release medium must be able to sustain pH stability over the course of the experiment. The osmolarity showed minimal change over the incubation period, with only minor increases observed over time. These increases could potentially result from sampling-related evaporation and/or the release of osmotically active substances from the polymer, such as monomeric units.^58^

For SBF and SSIF, temperature-dependent precipitation was observed from day 6 onward at 25 and 34 °C. As only SSIF contains HSA, the precipitation cannot be solely attributed to protein instabilities. This aligns with prior reports on the instability of complex ionic buffers at physiological temperatures, where calcium phosphate precipitation and pH drift limit their suitability for extended incubations.^40^ Despite the greater stability of 100 mM PB, it lacks the physiological complexity of SBF and SSIF, particularly with regard to mimicking ISF through the inclusion of proteins such as HSA. In the case of DEX, which exhibits approximately 50% protein binding in the presence of 2% HSA, the inclusion of proteins in the release medium could significantly influence the observed release profile.^59^ However, this aspect was disregarded in the present study, as no long-term stability was achieved. Consequently, 100 mM PB was selected for all studies to ensure the necessary methodological robustness. In addition to buffer composition, parameters, such as ISF flow, bulk volume, and tissue/ECM firmness, must be considered, as they influence polymer swelling and thus drug release. A 0.5% agarose gel was chosen because it allows diffusion of small molecules and mimics the bulk viscoelastic behavior of soft connective tissues, ECM-like hydrogels^60,61^ and, to a certain extent, the effects of mechanical compression.^45^ Additionally, agarose gels form pore sizes like those encountered in physiological tissues.^62^ When comparing the obtained G′ values of the 0.5% agarose gel (∼10^2^–10^3^ Pa) to SC adipose tissue and other soft connective tissues (G′ ∼ 10^3^–10^5^ Pa^63–66^ ), our gel is less stiff. However, its stiffness aligns closely with values reported for adipose-derived ECM substitutes and decellularized matrix hydrogels (G′ ∼ 10^2^–10^3^ Pa^64^). Although the implant is adjacent to the adipocytes, the functional environment is dominated by the ECM, which is why a comparison with the ECM, and thus also the simulation of the ECM, is more accurate.

Swelling of the implant was found to be lower in the Sotax setup compared to both static setups. The reduced swelling can be explained by hydrodynamic effects. Under flow, convective transport rapidly removes ions, degradation products, and other osmolytes released from the implant, thereby preventing the local accumulation of drug and other solutes. This process decreases the local osmotic pressure and thereby diminishes the driving force for water influx. The flow not only reduces the thickness of the unstirred boundary layer^67^ but might also mechanically restrict the expansion of the swollen network and further limit water penetration. Interestingly, swelling in agarose gels was comparable to static incubation, which contrasts with previous reports describing a reduction of swelling in agarose matrices.^45,46,48^ This discrepancy is likely related to differences in the polymer type (e.g., PLGA^45,46,48^ versus the present copolymer) and in the gels’ properties, as stronger agarose networks can more effectively constrain implant expansion. For this multiblock copolymer, the hydrophilic PEG domains mainly drive the water uptake and swelling, while the more hydrophobic PGLLA domains maintain the structural integrity and act as physical cross-linkers.^37,68,69^ The structure of the polymers of the SynBiosys platform is based on regulatory-accepted building blocks, which allow for a strategic modification of the composition and drug release.^38^ These polymers have already been used for the preparation of various dosage forms^37,38,68,70^ as they offer the advantage that, through the swelling of the polymer, acidic degradation products such as lactic and glycolic acids can be gradually removed from the matrix. This contrasts with the commonly used PLGA, which only shows swelling after distinct polymer degradation, resulting in an accumulation of acidic byproducts within the polymer matrix.^37^

For the interpretation of the release studies, we focused on the first 8 weeks, as Brandl et al. showed that release was diffusion dominated during this period, while degradation-dominated release occurred only after approximately 11 weeks.^37^ The cumulative release profiles demonstrated that the implant designs and test setups significantly affect the drug release performance. In the dynamic Sotax setup, a higher cumulative release of DEX was observed for both implant types compared to that in the gel setup, with a particularly higher drug release within the initial phase (first 3 days). This effect can be attributed to the fact that, in the Sotax setup, the implants were in direct contact with the release medium, and the released DEX molecules were continually removed. By contrast, for the gel setup, released DEX migrated through the gel before reaching the release medium, where it was finally detected; consequently, the drug concentration in the surrounding buffer was below the LLOQ on the first day. The presence of DEX within the agarose gel after 8 weeks supports this hypothesis, as significantly higher concentrations compared to the surrounding buffer system were found. This highlights the contribution of the gel matrix to the overall drug retention through the additional barrier, which is in accordance with previous studies reporting a lower drug release when embedded in agarose gels.^23,45^

When comparing the dynamic Sotax setup to previously published static release data, no significant differences in drug release were observed for the HME implant (100% infill). This suggests that for dense, nonporous implant structures, drug release is primarily governed by diffusion through the polymer matrix, with minimal influence from external flow or shear forces. In contrast, the FFF implant with a 25% infill exhibited a higher relative release under dynamic conditions. The continuous flow in the Sotax setup likely reduces the liquid boundary layer and thus^71^ maintains local sink conditions at the implant surface. Drug diffusion is facilitated, and local drug accumulation at the implant surfaces is prevented through higher convective forces. This effect can be observed more for the FFF implant due to the implant’s porous architecture, which offers an increased surface-area-to-volume ratio compared to a dense implant with the same outer dimensions.^37^ When comparing our release methods with existing literature, a significant lack of standardization in SC implant testing is revealed. While static models are used to some extent,^72^ the most prevalent method remains bulk fluid release under agitation. However, inconsistencies in media, volumes, and agitation speeds (40–150 rpm) hinder cross-study comparability.^47,62,73–76^ Furthermore, while the USP 4 is an established dynamic protocol for several SC formulations,^22,36,77,78^ its application for SC implants remains limited.

The Korsmeyer–Peppas model was applied to analyze the drug release behavior from the implants, as it is frequently used for polymer-based systems with limited swellability.^79–84^ One key limitation of this model is that it is valid only up to 60% cumulative drug release,^50^ which was not exceeded in any of the release profiles over the 8 weeks study period. Due to the differing infill densities of the HME (100%) and FFF (25%) implants, while maintaining the same outer dimensions and relative drug loading, the absolute DEX content varied. This difference accounts for the significantly higher relative release observed from the FFF implants and the approximately threefold higher *k*-values for the FFF implants obtained in the Korsmeyer–Peppas model, which also reflects the different structural and geometrical properties of the implant manufactured via FFF compared to that manufactured by HME. It is important to note that *k* is influenced not only by the solute’s diffusion coefficient and the macromolecular network properties but also by the geometry of the system,^49,50^ which limits direct comparability of the obtained *k*-values between implants with different infill designs. In contrast, the *n*-value from the model provides insight into the underlying release mechanism and is more commonly used for qualitative comparisons.^49^ For cylindrical samples such as ours, the critical threshold for Fickian diffusion is *n* = 0.45 rather than 0.5 (which applies to slabs).^50,84^ Given the implant dimensions (diameter/length = 2/40 = 0.05), which is below the critical 0.2 ratio, we assume predominantly one-dimensional diffusion, further justifying the use of the adapted *n* threshold.^50,81^ All implant types showed two distinct release phases (Table 3). In the Sotax setup, the initial phase (0.25–2 days) corresponded to a burst release, likely caused by readily available surface-associated drug,^45^ consistent with high daily release rates and previously published static release data by Brandl et al.^37^ For the second phase (3–56 days), *n*-values for both implant types (HME = 0.538, FFF = 0.491) were close to 0.45, indicating Fickian diffusion as the primary release mechanism alongside polymer swelling. The interpretation of the observed *n*-values is in line with the findings of other groups on, e.g., the drug release from cylindrical 3D-printed PCL/PEG implants.^85^ In general, *n*-values >0.45 are frequently reported for such geometries and are typically interpreted as being either predominantly Fickian diffusion-controlled (when close to 0.45) or a combined mechanism of diffusion and polymer swelling.^85,86^ The HME implants displayed an *n*-value of 0.538, closely matching the static reference value (0.53), confirming that hydrodynamic flow had little influence on drug release from dense, nonporous implants. In contrast, the FFF implants showed slightly higher *n*-values in the dynamic setup (0.491 vs 0.47 in static), in line with the observed differences in relative release and suggesting that flow conditions may affect porous structures more prominently. In the gel setup, the initial release phase was delayed (1–7 days), likely due to the additional diffusion barrier created by the agarose matrix. During this phase, *n*-values >1 were observed, typically indicating Super Case II transport. However, this is attributable to the initial diffusion barrier formed by the surrounding gel, which delays the measurable appearance of the drug in the adjacent buffer solution and thereby alters the observed release profile. The consistently higher *n*-values observed in the later phases compared to the Sotax and static setups indicate a combined mechanism of Fickian diffusion and Super Case II transport, which includes mechanisms such as polymer swelling and relaxation. This contrasts with the observed swelling behavior of the implants, which was similar in both the static and gel setups. However, the interpretation of the obtained fitting data should be made with caution, as the presence of the agarose gel introduces a multiphase diffusion system, which is additionally dependent on the agarose layer thickness. The equilibration of the drug within the gel over time complicates mechanistic conclusions and may overestimate the contribution of polymer dynamics to the overall release behavior. Consequently, the calculated *n*-values do not solely depict the underlying release mechanism of the implant itself. Instead, they reflect a composite release profile where the *n*-value is heavily influenced by the mass transport kinetics from the surrounding gel matrix into the sampling medium, i.e., 100 mM PB. Therefore, these parameters should not be interpreted as intrinsic polymer or implant characteristics, but rather as system-dependent values inherent to the specific experimental setup. For all release data, the *R* ^2^ ≥ 0.995 indicates a good fit of the model within all time frames, which is comparable to reported Korsmeyer–Peppas fitting data in the literature.^49,80,81^ In summary, this study provides important insights that should be considered when selecting an in vitro dissolution medium, particularly for long-term studies. It also sheds light on how ISF flow and tissue/ECM firmness influence the drug release behavior specifically of porous implants. It should be noted that a limitation of this study is the lack of in vivo validation of these parameters.

## 5 Conclusion

In conclusion, this study provides valuable insights into the interplay among implant design, test setup, and release medium characteristics, offering a framework for improving long-term in vitro drug release testing of SC implants. PB (100 mM) was selected as the release medium due to its superior stability for long-term studies compared to more physiological media such as SBF and SSIF, including HSA, which showed precipitation and protein degradation. Significant differences in release profiles and kinetic parameters were observed between the dynamic Sotax setup simulating ISF flow and the gel-based setup mimicking SC tissue/ECM firmness, compared to previously published static release data. The implant microstructure was identified as a key determinant of both the release profiles and their comparability across different setups. Open porous structures showed higher sensitivity to flow conditions, whereas dense, nonporous systems showed diffusion-dominated release that was largely unaffected by flow. Overall, this work underscores the importance of tailoring in vitro methods to the specific characteristics of SC drug delivery systems to enable more accurate drug release testing, particularly for long-term studies.

## Supplementary Material



## Notes

pH and osmolarity values of the release media, including statistical analysis; additional experimental details and results for drug-protein binding data; additional figure directly comparing HME and FFF implants for each release method (PDF)
