# Supplementary material for: Dynamic versus Static In Vitro Drug Release Testing of Subcutaneous Implants with Distinct Microstructures
Source: ACS Omega. 2026 Jul 8;11(28):41989–2000. doi: 10.1021/acsomega.6c02143 (PMC13393397; doi:10.1021/acsomega.6c02143)
Supplement: Supplementary file 1 [file ao6c02143_si_001.pdf]

Supplementary Information

Dynamic versus Static In Vitro Drug Release Testing of Subcutaneous Implants with Distinct Microstructures

Scarlett Zeiringer[1,†], Laura Wiltschko[1,†], Bianca Brandl[1,2], Anbu Palanisamy[3], Thanh Nguyen[3], Matthias Katschnig[4], Martin Spoerk[2,5], Simone Eder[2], Eva Roblegg[1,*]

[1] University of Graz, Institute of Pharmaceutical Sciences, Department of Pharmaceutical Technology and Biopharmacy, Universitätsplatz 1, 8010 Graz, Austria

[2] Research Center Pharmaceutical Engineering GmbH, Inffeldgasse 13, 8010 Graz, Austria

[3] InnoCore Pharmaceuticals, L.J. Zielstraweg 1, 9713 GX Groningen, The Netherlands

[4] Hage3D GmbH, Kratkystraße 2, 8020 Graz, Austria

[5] Institute of Process and Particle Engineering, Graz University of Technology, Inffeldgasse 13, 8010 Graz, Austria

[†]These authors contributed equally to this work.

*Correspondence:

Eva Roblegg
eva.roblegg@uni-graz.at
+43 316 380 8888

Buffer Characterization

Table S1: Mean pH values of phosphate buffer (PB) 100 mM, simulated body fluid (SBF) and simulated subcutaneous interstitial fluid (SSIF) over seven days upon polymer incubation in two distinct setups (i.e., 3 ml setup with buffer exchange and 10 ml setup without buffer exchange). The control setup represents solely buffer without incubated polymer.

pH	3 ml setup	10 ml setup	control
PB	7.36 ± 0.03<br>CV = 0.45%	7.31 ± 0.04<br>CV = 0.48%	7.32 ± 0.02<br>CV = 0.30%
SBF	7.50 ± 0.05<br>CV = 0.69%	7.42 ± 0.05<br>CV = 0.63%	7.42 ± 0.04<br>CV = 0.49%
SSIF	7.63 ± 0.08<br>CV = 1.10%	7.44 ± 0.06<br>CV = 0.79	7.45 ± 0.04<br>CV = 0.48%

Table S2: Simple linear regression analysis of measured pH values across all buffer systems (i.e., phosphate buffer 100 mM (PB), simulated body fluid (SBF) and simulated subcutaneous interstitial fluid SSIF)) and experimental setups over a 7-day period.

pH	3 ml setup	10 ml setup	control
PB	Slope = -0.009779<br>(95% CI: -0.01561 to -0.003951)<br>p = 0.0020<br>Deviation from zero = significant<br>R^2 = 0.3332	Slope = -0.001977<br>(95% CI: -0.008478 to 0.004524)<br>p = 0.5367<br>Deviation from zero = not significant<br>R^2 = 0.01545	Slope = 0.002543<br>(95% CI: -0.001441 to 0.006529)<br>p = 0.2006<br>Deviation from zero = not significant<br>R^2 = 0.06464
SBF	Slope = -0.01427<br>(95% CI: -0.02355 to -0.004992)<br>p = 0.0041<br>Deviation from zero = significant<br>R^2 = 0.2957	Slope = -0.01179<br>(95% CI: -0.01901 to -0.004559)<br>p = 0.0025<br>Deviation from zero = significant<br>R^2 = 0.3109	Slope = -0.007861<br>(95% CI: -0.01381 to -0.001910)<br>p = 0.0117<br>Deviation from zero = significant<br>R^2 = 0.2284
SSIF	Slope = -0.02069<br>(95% CI: -0.03629 to -0.004916)<br>p = 0.0122<br>Deviation from zero = significant<br>R^2 =0.2344	Slope = -0.02240<br>(95% CI: -0.02813 to -0.01667)<br>p < 0.0001<br>Deviation from zero = significant<br>R^2 = 0.7218	Slope = -0.01353<br>(95% CI: -0.01719 to -0.009873)<br>p < 0.0001<br>Deviation from zero = significant<br>R^2 = 0.6987

Table S3: Mean osmolarity of phosphate buffer (PB) 100 mM, simulated body fluid (SBF) and simulated subcutaneous interstitial fluid (SSIF) over seven days upon polymer incubation in two distinct setups (i.e., 3 ml setup with buffer exchange and 10 ml setup without buffer exchange). The control setup represents solely buffer without incubated polymer.

Osmolarity	Start (osmol/L)	3 ml setup	10 ml setup	control
PB	0.223 ± 0.001	0.253 ± 0.017<br>(+13.96 ± 7.64%)	0.248 ± 0.019<br>(+11.18 ± 8.07%)	0.246 ± 0.006<br>(+10.47 ± 2.53%)
SBF	0.355 ± 0.011	0.437 ± 0.028<br>(+24.53 ± 7.62%)	0.448 ± 0.006<br>(+25.44 ± 5.88%)	0.450 ± 0.011<br>(+25.83 ± 4.79%)
SSIF	0.354 ± 0.020	0.436 ± 0.010<br>(+34.15 ± 0.13%)	0.443 ± 0.012<br>(+20.84 ± 3.59%)	0.440 ± 0.023<br>(+19.69 ± 6.61%)

Table S4: Simple linear regression analysis of measured osmolarity across all buffer systems (i.e., phosphate buffer 100 mM (PB), simulated body fluid (SBF) and simulated subcutaneous interstitial fluid SSIF)) and experimental setups over a 7-day period.

Osmolarity	3 ml setup	10 ml setup	control
PB	Slope = 0.006127<br>(95% CI: 0.003782 to 0.008472)<br>p < 0.0001<br>Deviation from zero = significant<br>R^2 = 0.5479	Slope = 0.003913<br>(95% CI: 0.001891 to 0.005934)<br>p = 0.0005<br>Deviation from zero = significant<br>R^2 = 0.3887	Slope = 0.003201<br>(95% CI: 0.002580 to 0.003823)<br>p < 0.0001<br>Deviation from zero = significant<br>R^2 = 0.8182

SBF	Slope = 0.01110<br>(95% CI: 0.008600 to 0.01359)<br>$p < 0.0001$<br>Deviation from zero = significant<br>$R^2 = 0.7782$	Slope = 0.01161<br>(95% CI: 0.008538 to 0.01468)<br>$p < 0.0001$<br>Deviation from zero = significant<br>$R^2 = 0.7078$	Slope = 0.01233<br>(95% CI: 0.009773 to 0.01490)<br>$p < 0.0001$<br>Deviation from zero = significant<br>$R^2 = 0.7973$
SSIF	Slope = 0.01157<br>(95% CI: 0.009203 to 0.01393)<br>$p < 0.0001$<br>Deviation from zero = significant<br>$R^2 = 0.8095$	Slope = 0.01022<br>(95% CI: 0.007108 to 0.01332)<br>$p < 0.0001$<br>Deviation from zero = significant<br>$R^2 = 0.6470$	Slope = 0.009666<br>(95% CI: 0.007318 to 0.01201)<br>$p < 0.0001$<br>Deviation from zero = significant<br>$R^2 = 0.7420$

Drug-protein binding to HSA

Materials and Methods

Drug-protein binding measurements were performed according to an adapted protocol by Wiltschko et al.[1] using a rapid equilibrium dialysis (RED) device with a single-use plate for 48 samples with 8 K MWCO inserts obtained from Thermo Scientific Pierce (Rockford, IL, USA). The tested samples comprised 2% HSA in PB, which was spiked with either 20 µg/ml or 50 µg/ml DEX, which are well within the range of the analysis method. In brief, 100 µl of the samples (n=3 per time point and concentration) were added to the plasma chamber, and 350 µl PB were added to the buffer chamber of the RED inserts. The plates were sealed with a polyester sealing tape and incubated at 34 °C while agitating at 150 rpm. First, the time required to obtain a steady state equilibrium between the plasma and the buffer chamber was evaluated. In the present case, the equilibrium was attained after 8 h. After this, samples from the plasma and buffer chamber were collected and directly prepared for the subsequent DEX quantification. The samples from the buffer chambers were analyzed directly using HPLC-UV (chapter 2.10) without additional sample preparation, while for the samples from the plasma chamber protein precipitation was performed. Samples were stored at -20 °C until further analysis.

To remove HSA from the RED plasma chamber samples prior to HPLC-UV measurements, 50 µl sample was spiked with 150 µl precipitation agents (90/10 ACN/MQ + 0.1% formic acid). The samples were then vortexed for 30 s and centrifuged at 4000 rpm and 4 °C for 15 min until a protein pellet was visible. 50 µl of the supernatant was then added to 50 µl of PB, transferred into HPLC vial inlets and the DEX content was then analyzed using HPLC-UV (chapter 2.10.). Additionally, for the HPLC analysis of the protein precipitation samples, a separate calibration was performed, with calibration samples ranging from 1 to 20 µg/ml in 2% HSA in PB.

The protein bound drug fraction was calculated from the obtained drug concentration in buffer and plasma chamber of the RED according to equation 1

$$\%bound\ drug\ fraction = 100 - \frac{c(buffer\ chamber)}{c(plasma\ chamber)} * 100$$

The recovery of each RED sample was calculated using equation 2

$$\%recovery = \frac{c(buffer\ chamber)*350\ \mu l + c(plasma\ chamber)*100\ \mu l}{c(sample)*100\ \mu l} * 100$$

Results and Discussion

Steady-state equilibrium between the plasma and buffer chamber was reached after 8 h of incubation, as no significant difference in the DEX concentration in the plasma chamber was observed between 8 h and 10 h ($p > 0.9999$). Consequently, an incubation time of 8 h was considered sufficient for the main study. 2% HSA/PB was used due to its superior stability. The protein-free fraction of DEX was 48.95 ± 2.17% at 20 µg/ml and 52.86 ± 2.05% at 50 µg/ml. As no significant difference between these values was found ($p = 0.1143$), it can be concluded that DEX does not exhibit concentration-dependent binding to HSA within this range. The recovery was 103.41 ± 0.96% for 20 µg/ml and 104.95 ± 2.81% for 50 µg/ml, which are well within the acceptable RED recovery range of 70-125%[2,3].

These protein binding findings are critical, as a ~50% bound fraction significantly alters the diffusion gradient and in vivo release kinetics by reducing the pharmacologically active, unbound drug available to cross capillary membranes. Therefore, it is of utmost importance to develop strategies in the future that take this into account. To the best of the authors' knowledge, no long-term drug release studies have yet been conducted in protein-containing release media, neither for DEX-loaded implants nor for other SC implant systems. Consequently, it remains unclear whether the use of such physiologically more relevant media could improve the in vitro - in vivo correlation (IVIVC). Additionally, long-term in vivo studies are rare or conducted in animal models that are not comparable to humans, making direct comparison difficult.

Drug release studies

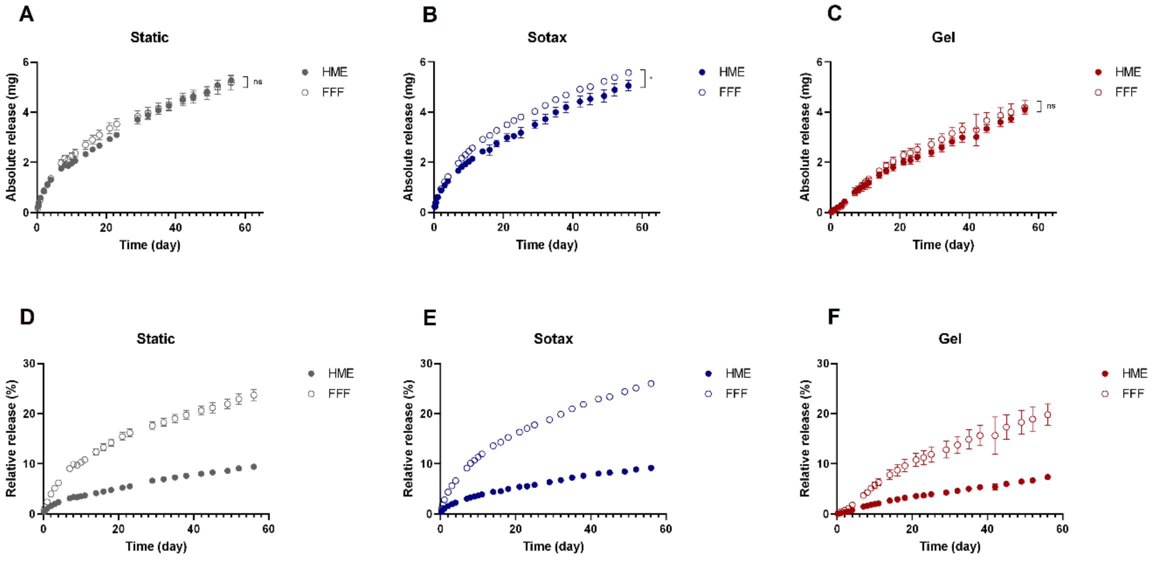

Figure S1: Drug release profiles of the Static, Sotax and Gel method comapring hot melt extrusion (HME) and fused filemant fabrication (FFF) implant over eight weeks. A static release setup by Brandl et al[4]. is inluded for comparision A) Absolute drug release (mg) in the static setup. B) Absolute drug release (mg) in the sotax setup. C) Absolute drug release (mg) in the gel setup. D) Relative release (%) in the static setup. E) Relative release (%) in the sotax setup. F) Relative release (%) in the gel setup. Significance was tested for the cumulative absolute drug release at day 56. Figure 1A – Static: HME vs FFF = ns (p=0.6388); Sotax: HME vs FFF = * (p=0.0183); Gel: HME vs FFF = ns (p=0.6513).

References

1. Wiltschko L, Roblegg E, Raml R, Birngruber T. Small volume rapid equilibrium dialysis (RED) measures effects of interstitial parameters on the protein-bound fraction of topical drugs. *J Pharm Biomed Anal*. 2023;234:115571. doi:10.1016/j.jpba.2023.115571

2. Di L, Breen C, Chambers R, et al. Industry Perspective on Contemporary Protein-Binding Methodologies: Considerations for Regulatory Drug-Drug Interaction and Related Guidelines on Highly Bound Drugs. *J Pharm Sci*. *Elsevier B.V.* 2017;106(12):3442-3452. doi:10.1016/j.xphs.2017.09.005

3. Waters NJ, Jones R, Williams G, Sohal B. Validation of a rapid equilibrium dialysis approach for the measurement of plasma protein binding. *J Pharm Sci*. 2008;97(10):4586-4595. doi:10.1002/jps.21317

4. Brandl B, Zeiringer S, Loidl L, et al. Dual-extruder 3D-printing of biodegradable subcutaneous implants for controlled drug delivery. *Addit Manuf*. 2025;110. doi:10.1016/j.addma.2025.104928
